# Gene expression profiling of white adipose tissue reveals paternal transmission of proneness to obesity

**DOI:** 10.1038/srep21693

**Published:** 2016-02-12

**Authors:** Sumiyo Morita, Kazuhiko Nakabayashi, Tomoko Kawai, Keiko Hayashi, Takuro Horii, Mika Kimura, Yasutomi Kamei, Yoshihiro Ogawa, Kenichiro Hata, Izuho Hatada

**Affiliations:** 1 Laboratory of Genome Science, Biosignal Genome Resource Center, Institute for Molecular and Cellular Regulation, Gunma University, 3-39-15 Showa-machi Maebashi, 371-8512, Japan; 2 Department of Maternal-Fetal Biology, National Research Institute for Child Health and Development, 2-10-1 Okura Setagaya-ku Tokyo, 157-8535, Japan; 3 Laboratory of Molecular Nutrition, Graduate School of Environmental and Life Science, Kyoto Prefectural University, 1-5 Hangi-cho, Shimogamo, Sakyo-ku, Kyoto, 606-8522, Japan; 4 Department of Molecular Endocrinology and Metabolism, Graduate School of Medical and Dental Sciences, Tokyo Medical and Dental University, 1-5-45 Bunkyo-ku, Yushima, Tokyo, 113-8510, Japan

## Abstract

Previously, we found that C57BL/6J (B6) mice are more prone to develop obesity than PWK mice. In addition, we analyzed reciprocal crosses between these mice and found that (PWK × B6) F1 mice, which have B6 fathers, are more likely to develop dietary obesity than (B6 × PWK) F1 mice, which have B6 mothers. These results suggested that diet-induced obesity is paternally transmitted. In this study, we performed transcriptome analysis of adipose tissues of B6, PWK, (PWK × B6) F1, and (B6 × PWK) F1 mice using next-generation sequencing. We found that paternal transmission of diet-induced obesity was correlated with genes involved in adipose tissue inflammation, metal ion transport, and cilia. Furthermore, we analyzed the imprinted genes expressed in white adipose tissue (WAT) and obesity. Expression of paternally expressed imprinted genes (PEGs) was negatively correlated with body weight, whereas expression of maternally expressed imprinted genes (MEGs) was positively correlated. In the obesity-prone B6 mice, expression of PEGs was down-regulated by a high-fat diet, suggesting that abnormally low expression of PEGs contributes to high-fat diet-induced obesity in B6 mice. In addition, using single-nucleotide polymorphisms that differ between B6 and PWK, we identified candidate imprinted genes in WAT.

Parent-of-origin effects, which arise when the phenotype of an allele depends on whether it is inherited from the father or mother, are often considered to reflect genomic imprinting. Prader–Willi syndrome, a well-known imprinting disorder, is the most common known genetic cause of morbid obesity in children^1^, and genetic studies of human^2^,^3^,^4^ and mouse^5^,^6^,^7^ indicate that genomic imprinting is involved in obesity. Obesity, a growing world-wide health problem, causes metabolic diseases such as type 2 diabetes, hypertension, and cardiovascular disease. Therefore, the mechanisms underlying inheritance of obesity are of great medical interest.

We previously showed that diet-induced obesity is paternally transmitted in the specific model used, i.e. using crosses between the two inbred lines, C57BL/6J (B6) and PWK^8^. C57BL/6J mice are prone to develop diet-induced obesity, whereas PWK mice are resistant. We analyzed offspring of reciprocal crosses between these strains, i.e., (PWK × B6) F1 and (B6 × PWK) F1. These mice are phenotypically distinct: F1 mice with B6 fathers ([PWK × B6] F1) are more sensitive to dietary obesity than F1 mice with PWK fathers ([B6 × PWK] F1), suggesting paternal transmission of diet-induced obesity. We also demonstrated an association between imprinted genes and obesity: In white adipose tissue (WAT), *Igf2* and *Peg3*, paternally expressed imprinted genes (PEGs) involved in the regulation of body fat accumulation, were down-regulated in B6 and (PWK × B6) F1 mice, which are susceptible to high fat diet (HFD)-induced obesity, but not in PWK and (B6 × PWK) F1 mice, which are resistant.

Diet-induced obesity affects expression in many tissues, but we first focused on expression in WAT because expression of an imprinted gene, *Igf2*, was significantly affected in WAT and was not significantly affected in other tissues [8, unpublished results]. In this study, we used next-generation sequencing to perform transcriptome analysis (RNA-seq) of adipose tissues of B6, PWK, (PWK × B6) F1, and (B6 × PWK) F1 mice. Specifically, we analyzed the differences in paternal and maternal allele-dependent gene expression in WAT in animals of all four genotypes exposed to control diet or HFD. We found that alterations in inflammation, metal ion transport (mitochondrial function), and cilia (hedgehog signaling) in adipose tissue resulting from diet-induced obesity were influenced by paternal, but not maternal, alleles.

Furthermore we analyzed the relationship between genomic imprinting and obesity. The expression of PEGs in WAT was negatively correlated with body weight, whereas expression of maternally expressed imprinted genes (MEGs) was positively correlated. The WAT gene expression profiles, which differed significantly between B6 and PWK mice, potentially explain why PWK mice are resistant to diet-induced obesity.

## Results

### Transcriptome analysis of adipose tissues

**Figure 1:**
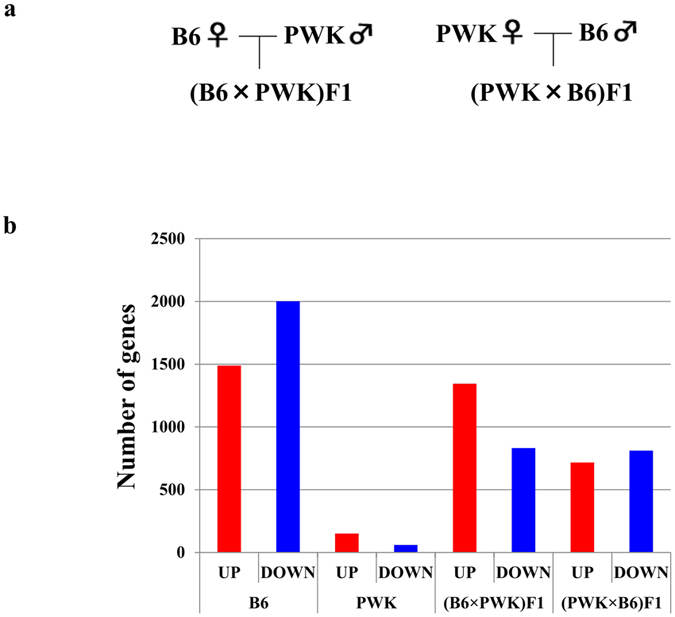
Number of differentially expressed genes (>2.0-fold) in WAT of mice fed a HFD vs. mice fed a control diet. (**a**) Generation of reciprocal crosses between B6 and PWK mice. (B6 × PWK) F1 is the offspring of a B6 mother and PWK father, and (PWK × B6) F1 is the offspring of a PWK mother and B6 father. (**b**) Number of genes shown by NGS analysis to be differentially expressed in WAT of HFD mice relative to WAT of mice fed a control diet. Red bars indicate the number of up-regulated genes (p < 0.05, >2-fold), and blue bars indicate the number of down-regulated genes (p < 0.05, >2-fold).

C57BL/6J (B6) mice, PWK mice, and the F1 progeny obtained by reciprocal crosses between these strains were fed either a normal control diet (Con) or an HFD for 15 weeks starting at the age of 6 weeks. Genome-wide expression analysis by next-generation sequencing was performed on WAT from each strain of mice (n = 3 for each strain/diet combination). A summary of the reads for each sample is presented in Table S1, including total reads and >Q30 reads. Q30 is Illumina’s quality score and is more reliable and less likely to be incorrect. The correlation coefficients in the same experimental groups are summarized in Table S2. We analyzed the expression levels of 15825 genes, the number of differentially expressed genes (p < 0.05, >2-fold), and the directions of expression changes are summarized in Fig. 1.

In WAT of B6 mice, 3490 genes were differentially expressed (1489 up-regulated, 2001 down-regulated) in HFD vs. Con, the most dramatic relative change among the four strains. By contrast, in WAT of PWK mice, only 211 genes were differentially expressed (151 up-regulated, 60 down-regulated) as a function of diet.

### Paternal dependence of gene expression changes in WAT

We previously showed that B6 mice are more sensitive to HFD-induced obesity than PWK mice, and that (PWK × B6) F1 mice, which have B6 fathers, are more sensitive to diet-induced obesity than (B6 × PWK) F1 mice, which have B6 mothers, suggesting paternal transmission of diet-induced obesity. Therefore, we analyzed the parental dependence of gene expression changes in WAT of these F1 mice.

**Figure 2:**
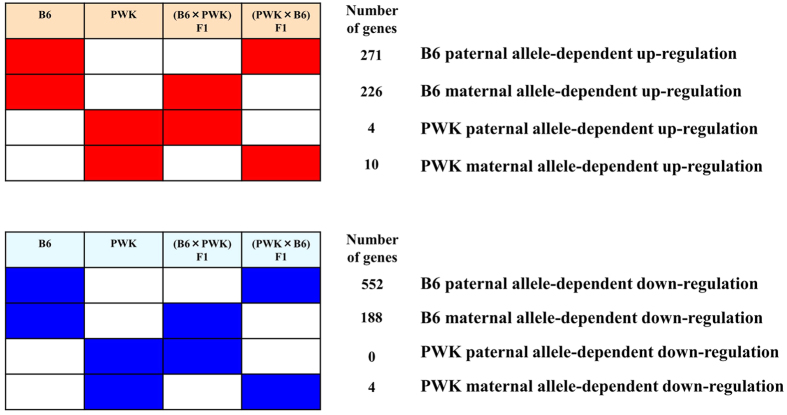
Number of genes with paternal or maternal-dependent expression changes in WAT. The upper panel shows up-regulated genes, and the lower panel shows down-regulated genes. For example, a total of 271 genes were significantly up-regulated (p < 0.05, >2.0-fold) in both B6 and (PWK × B6) F1, but not in PWK or (B6 × PWK) F1; these were defined as B6 paternal allele-dependent up-regulated genes. By contrast, 226 genes were significantly up-regulated (p < 0.05, >2.0-fold) in both B6 and (B6 × PWK) F1, but not in PWK and (PWK × B6) F1; these were defined as B6 maternal allele-dependent up-regulated genes.

We identified 271 genes that were significantly up-regulated (p < 0.05, >2-fold) in B6 and (PWK × B6) F1, which developed diet-induced obesity, but not in PWK and (B6×PWK) F1, which did not become obese (B6 paternal allele-dependent up-regulated genes. Fig. 2). We also identified 552 B6 paternal allele-dependent down-regulated genes (p < 0.05, >2-fold) (Fig. 2). In addition, we identified 226 B6 maternal allele-dependent up-regulated genes and 188 B6 maternal allele-dependent down-regulated genes (Fig. 2). In PWK mice, only a few genes differed between HFD and Con (Fig. 1); therefore, in subsequent analysis, we focused on B6 parental allele-dependent up- or down-regulated genes.

Next, we investigated the biological characteristics of these parental allele-dependent genes using DAVID (http://david.abcc.ncifcrf.gov/), a functional classification tool^9^,^10^. B6 paternal allele-dependent up-regulated genes were significantly associated with Gene Ontology (GO) biological process (BP) annotation terms related to immune responses, such as leukocyte activation, myeloid leukocyte activation, and mast cell activation (Table 1a and Table S3). These genes were also significantly associated with KEGG pathways such as “natural killer cell mediated cytotoxicity” and “chemokine signaling pathway” (Table S3). The B6 paternal allele-dependent down-regulated genes were significantly associated with GO BP terms related to ion transport and GO cellular component (CC) terms related to cell projection (cilia) (Table 1b, Table S4). Cilia may be involved in adipocyte differentiation, as described in the Discussion. By contrast, the B6 maternal allele-dependent up-regulated genes were significantly associated with GO terms related to the cell cycle (Table 2 and Table S5), whereas the B6 maternal allele-dependent down-regulated genes were not specifically associated with any GO term (Table S6).

### Correlation between body weight and the expression of PEGs and MEGs

**Figure 3:**
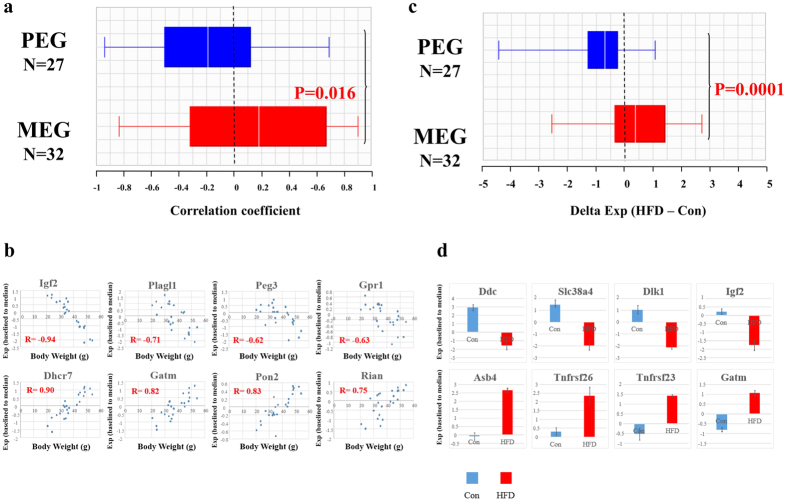
Correlation between body weight and expression of PEGs and MEGs. (**a**) Correlation between body weight and expression of 27 PEGs and 32 MEGs in WAT, presented as box plots. (**b**) Examples of correlation between body weight and expression of PEGs or MEGs. *Igf2*, *Plagl1*, *Peg3*, and *Gpr1* are PEGs, and *Dhcr7*, *Gatm*, *Pon2*, and *Rian* are MEGs. Expression were baselined to the median of each gene. (**c**) Differential expression of 27 PEGs and 32 MEGs between HFD and control diet B6 mice (HFD – Con). (**d**) Examples of differential expression of imprinted genes between HFD and control diet B6 mice. Expression of HFD and control diet B6 mice were presented. Expression were baselined to the median of each gene. *Dbc*, *Slc38a*, *Dlk1*, and *Igf2* are PEGs, and *Asb4*, *Tnfrsf26*, *Tnfrsf23*, and *Gatm* are MEGs.

We previously demonstrated a negative correlation between paternal transmission of HFD-induced obesity and expression of two PEGs, *Igf2* and *Peg3*, suggesting that these genes might contribute to fat accumulation, or the symptoms associated with obesity^8^. To systematically elucidate the connection between HFD-induced obesity and genomic imprinting, we analyzed the relationship between obesity and the expression of known imprinted genes (93 genes, including 40 PEGs and 53 MEGs) in WAT transcriptome data. Among the 93 imprinted genes, 60 (27 PEGs and 32 MEGs) were expressed in WAT. We calculated the correlation coefficient between body weight and the expression of each imprinted gene in WAT of each strain of mice (n = 3 for each strain/diet combination). Surprisingly, expression of PEGs tended to be negatively correlated with body weight, whereas expression of MEGs tended to be positively-correlated (Fig. 3a), suggesting that PEGs exert anti-obesity effects whereas MEGs promote obesity. Fig. 3b shows examples of correlations between body weight and expression of PEGs or MEGs. We also systematically analyzed HFD-induced expression changes of imprinted genes in obesity-prone B6 mice. If PEGs exert anti-obesity effects, they should be down-regulated in HFD-fed B6 mice. Consistent with this prediction, expression of PEGs was down-regulated by an HFD, whereas expression of MEGs was up-regulated (Fig. 3c).

### Screening for new imprinted genes in WAT

**Figure 4:**
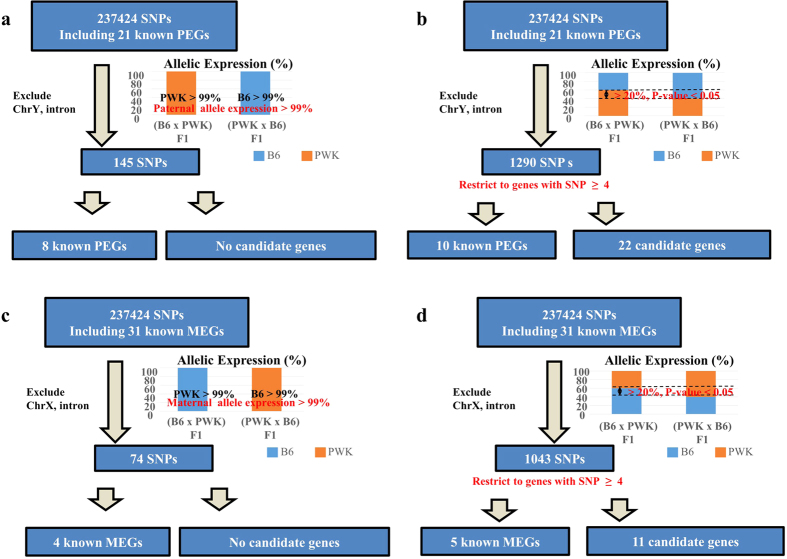
Screening for PEGs and MEGs in WAT. (**a**) Strict selection criteria for identification of new PEGs. (**b**) Mild selection criteria for identification of new PEGs. (**c**) Strict selection criteria for identification of new MEGs. (**d**) Mild selection criteria for identification of new MEGs.

**Figure 5:**
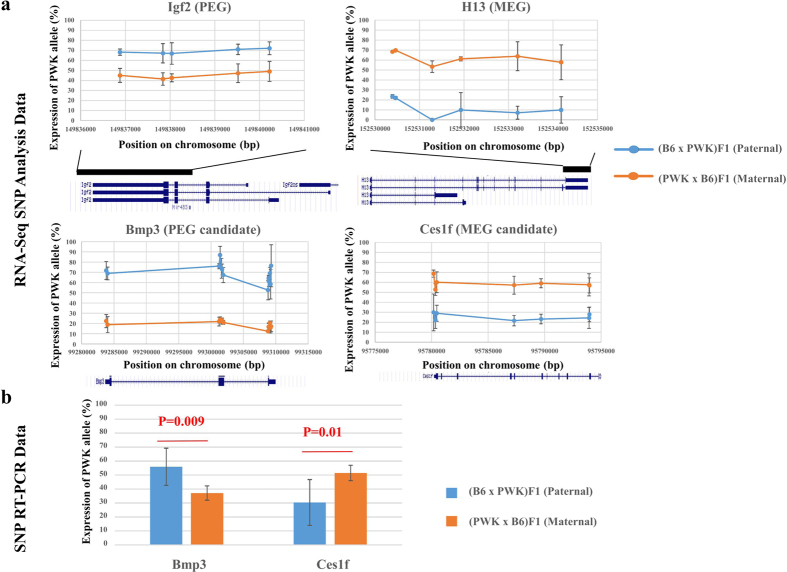
PWK allele expression at SNP loci. (**a**) PWK allele expression at several SNP loci in known imprinted genes (*Igf2*, *H13*) and candidate imprinted genes (*Bmp3*, *Ces1f*). The map of each locus is provided beneath the graph. (**b**) Ratio of expression (B6 allele: PWK allele) of candidates. These results were confirmed by RT-PCR followed by digestion with restriction enzymes whose recognition sites contained SNPs between B6 and PWK.

To date, about 150 imprinted genes have been identified. We tried to identify new imprinted genes expressed in WAT. Using single-nucleotide polymorphisms (SNPs) that differ between B6 and PWK detected in our RNA-seq data, we determined whether genes were expressed from the B6 or PWK allele in WAT of (B6 × PWK) F1 and (PWK × B6) F1 mice. Using the AVADIS NGS software, we extracted 237,424 expressed SNPs that differ between B6 and PWK. After excluding SNPs on the Y chromosome, SNPs not in identified exons, and SNPs with a small number of reads (average total number of reads <10, at least one mouse with no read), we used the remaining SNPs to further screen imprinted genes. To screen for PEGs, we initially selected SNPs for which the ratio of paternal allele expression was over 99% in both (B6 × PWK) F1 and (PWK × B6) F1. This set of 145 SNPs, included eight known PEGs, but did not include any candidate imprinted genes (strict selection, Fig. 4a). Therefore, we subsequently applied a milder selection criterion: (1) paternal allele expression was significantly higher than maternal allele expression (Student’s t-test, p < 0.05), (2) paternal allele expression was over 50% and maternal allele expression was under 50%, and (3) paternal allele expression was at least 20% higher than maternal allele expression. We selected 1290 SNPs, and then selected genes that included at least four of these SNPs. The resultant list of genes included 10 known and 22 candidate PEGs (mild selection, Fig. 4b, Table S7). We also screened for MEGs using the same criteria, yielding a list of 11 candidate MEGs (Fig. 4c,d, Table S8). Table 3 lists the candidate imprinted genes. Figure 5a shows examples of PWK allele expression at several SNP loci of known imprinted genes (*Igf2*, a PEG, and *H13*, an MEG) and candidate imprinted genes (*Bmp3*, a PEG, and *Ces1f*, an MEG). We confirmed these results by RT-PCR followed by digestion with restriction enzymes whose recognition sites contain SNPs between B6 and PWK (Fig. 5b). Thus, we were able to identify candidate imprinted genes in WAT by screening using the mild selection criteria.

### Comparison of gene expression in B6 and PWK

**Figure 6:**
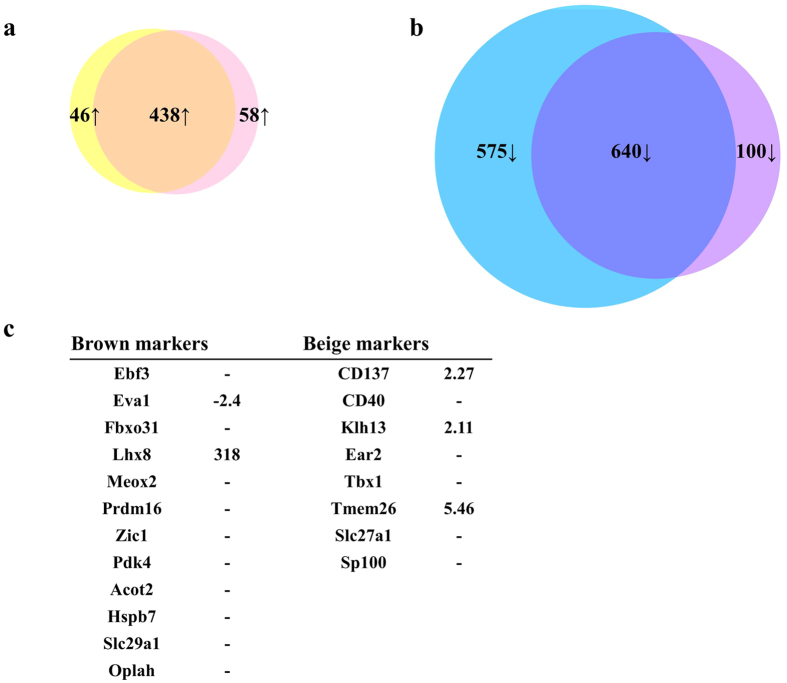
Venn diagram of differentially expressed genes (>5.0-fold) in WAT of PWK fed a control diet or HFD, relative to WAT of B6 mice fed a control diet. (**a**) Yellow circle indicates genes up-regulated in WAT of PWK fed a control diet relative to WAT of B6 fed a control diet (p < 0.05, >5.0-fold). Pink circle indicates genes up-regulated in WAT of PWK fed a HFD relative to WAT of B6 fed a control diet (p < 0.05, >5.0-fold). (**b**) Blue circle indicate genes down-regulated in WAT of PWK fed control diet relative to WAT of B6 with control diet (p < 0.05, >5.0-fold). Purple circle indicates genes down-regulated in WAT of PWK fed a HFD relative to WAT of B6 fed a control diet (p < 0.05, >5.0-fold). (**c**) Assessment of mouse brown and beige adipocyte markers in WAT of PWK and B6 mice. Numbers indicate fold change in expression in WAT of PWK relative to WAT of B6. (−) indicates no significant difference in expression between PWK and B6.

We previously showed that PWK mice are resistant to obesity and exhibit drastic differences in glucose clearance relative to B6. To understand the mechanisms underlying resistance to obesity in PWK, we compared WAT gene expression in B6 and PWK mice. Specifically, we compared the WAT expression profile between PWK fed a control diet, PWK fed a HFD, and B6 fed a normal diet. A total of 438 genes were up-regulated (p < 0.05, 5-fold) and 640 genes were down-regulated (p < 0.05, 5-fold) in WAT of PWK fed either diet relative to WAT of control-fed B6 (Fig. 6a,b). Functional annotation clustering and GO analysis of the up-regulated genes revealed a significant association with the annotation term “immunoglobulin-like” (Table S9), whereas down-regulated genes were significantly associated with terms such as “extracellular region”, “plasma membrane”, and “MHC protein complex” (Table 4, Table S10).

Among the 438 up-regulated genes was *Ucp1*, which is mainly expressed in brown adipose tissue (BAT); the UCP protein uncouples substrate oxidation from ATP production, resulting in thermogenesis^11^. *Ucp1* was dramatically up-regulated (378-fold) in WAT of PWK relative to WAT of B6. Recently, UCP1-expressing thermogenic adipocytes were identified in WAT; these cells are termed ‘brite’^12^ or ‘beige’^13^ adipocytes. Hence, we investigated whether these novel adipocytes were present in WAT of PWK. To this end, we examined the expression of well-established rodent brown and beige adipocyte markers^12^,^13^ in WAT of PWK and B6 mice (Fig. 6c). The brown marker Lhx8 was highly up-regulated (318-fold), and the beige markers CD137, Klh13, and Tmem26 were more modestly up-regulated, in PWK relative to B6.

## Discussion

### Paternal alleles influence inflammation and adipocyte differentiation in diet-induced obesity

In this study, we used next-generation sequencing to perform transcriptome analysis of WAT of B6, PWK, (PWK × B6) F1, and (B6 × PWK) F1 fed a control or high-fat diet (HFD), and analyzed the paternal dependency of gene expression changes in WAT of the F1 mice. Paternal transmission of diet-induced obesity was positively associated with genes involved in immune responses, and negatively associated with genes involved in ion transport and cell projection (cilium). Recent work showed that obesity is associated with chronic low-grade inflammation, which exerts profound effects on metabolic pathways and contributes to development of glucose tolerance and insulin resistance^14^,^15^,^16^,^17^. Adipose tissue in obese subjects is characterized by macrophage infiltration and the production of inflammatory mediators. In this study, expression levels of the macrophage marker CD68, as well as TNF-alpha and CCL2 (MCP1), were up-regulated in B6 and (PWK × B6) F1, but not in PWK or (B6 × PWK) F1 (data not shown). HFD feeding increases the amount and activity of many types of immune cells, including macrophages as well as mast cells, neutrophils, and T and B lymphocytes^18^. Our functional annotation data revealed that genes associated with immune responses or activation of immune cells such as myeloid and lymphoid cells (T-cell and B-cell activation) were up-regulated in B6 and (PWK × B6) F1, but not in PWK or (B6 × PWK) F1, suggesting that immune responses or immune cell activation leading to chronic low-grade inflammation are caused by paternal alleles.

We also showed that expression of genes associated with ion transport and cell projection (cilium) was negatively correlated with paternal transmission of diet-induced obesity. Cilia, which function as sensory antennae, are involved in the regulation of a number of key cellular signaling pathways, including hedgehog signaling^19^,^20^. Hedgehog signaling reduces expression of PPARG and CEBPA in 3T3-L1 cells, and is thus anti-adipogenic^21^,^22^. Therefore, reduced expression of cilia could inhibit hedgehog signaling, thereby increasing white fat mass. Notably in this regard, clinical manifestations of cilia are associated with morbid obesity, such as Bardet-Biedl syndrome^23^,^24^,^25^ and Alström syndrome^26^. Thus, paternal transmission of diet-induced obesity could be attributed to obesity-induced inflammation and activation of adipogenesis.

Maternal dependency of gene expression differed strikingly from paternal dependency. Maternally transmitted B6 alleles were positively associated with the cell cycle, suggesting that maternal allele-dependent up-regulated genes are involved in cell cycle progression. Many genes associated with the cell cycle influence metabolism and obesity^27^. However, our results suggest that the paternal allele dependent up-regulation of inflammation genes and down-regulation of cilia genes exerts a greater effect on diet-induced obesity than maternal allele-dependent up-regulation of cell cycle genes.

Not only imprinted genes, but also non-imprinted genes can contribute to paternal transmission of HFD-induced obesity. Mott *et al.*^34^ recently reported that a large proportion of quantitative traits in mice display parent-of-origin effects by interaction with imprinted loci and deduced that the importance of the number of imprinted genes is secondary to their interactions. This study implies that non-imprinted genes influence parent-of-origin effects by dysregulating the expression of imprinted genes.

### Evolutionary significance of the effects of PEGs and MEGs on obesity

We previously demonstrated a negative correlation between paternal transmission of HFD-induced obesity and expression of two imprinted genes, *Igf2* and *Peg3* (paternal expressed gene, PEG). Hence, in this study we analyzed expression of imprinted genes in WAT. The expression of PEGs was negatively correlated with body weight, whereas expression of MEGs was positively correlated. David Haig proposed the kinship theory of genomic imprinting^28^,^29^,^30^,^31^,^32^, which notes that maternally and paternally inherited alleles are differentially related to the kin of the fetus. Kinship theory predicts that maternal alleles favor lower levels of maternal investment than paternal alleles, because maternal alleles are necessarily present in the mother and reduced investment confers a strong advantage on the mother, whereas the opposite is true for paternal alleles. This theory may also apply to obesity: if adiposity of children reduces the requirement for maternal provisioning, the mother may benefit from having to allocate a lower proportion of limited food to their offspring during times of famine; thus MEGs may favor greater fat-reserve development in children^33^. By contrast, PEGs may favor lower fat-reserve development because offspring benefit from receiving more food from the mother, and reducing maternal investment confers no advantage on the father. Thus, MEGs tend to be pro-obesity genes whereas PEGs tend to be anti-obesity genes. The findings of this study support the extension of Haig’s theory to obesity and help to explain the evolution of obesity in the mammals. In obesity-prone B6 mice, the expression of PEGs is down-regulated by a HFD (Fig. 3c), suggesting that abnormally low expression of PEGs contributes to HFD induced obesity in this strain.

Further functional studies of the roles of imprinted genes in the transmission of obesity should be conducted in future.

### Identification of candidate imprinted genes

To date, around 150 imprinted genes have been identified. We screened for candidate imprinted genes using RNA-seq data from WAT of F1 male progeny obtained by reciprocal crosses between two parental strains. We did not discover any new completely imprinted genes, i.e., genes that only express the paternal or maternal allele. However, we did find several partially imprinted genes that were predominantly expressed from either the paternal or maternal allele, but were also expressed to a lesser extent from the other allele (Figs 4 and 5). Indeed, several known imprinted genes are also partially imprinted, e.g., *Igf2* and *H13* (Fig. 5a). Therefore, our mild selection approach was suitable for identification of new imprinted genes. None of the candidate imprinted genes were located near known imprinted genes, with the exception of *Cobl*, a neighbor of a known MEG. *Ank2*, *Arhgef37*, and *Slc16a12* are located in germline differentially methylated regions^35^, which are CpG islands lying outside the known imprinting control regions that are differentially methylated in oocytes and sperm. Approximately half of these differentially methylated regions appear to be at least partially resistant to the global DNA demethylation that occurs during preimplantation development^35^.

### Functional significance of differentially expressed genes in B6 and PWK

PWK mice are more resistant to obesity than B6 mice, and glucose clearance in PWK fed either a control diet or HFD is superior to that in B6 mice. Furthermore, our results showed that the immune responses of PWK mice differed significantly from those of B6 mice. In particular, genes associated with antigen processing and presentation, such as major histocompatibility complex (MHC) I and MHC II, were down-regulated in PWK. A previous report showed that obesity is associated with elevated expression of MHC II, an important determinant of HFD-induced obesity, in primary human and mouse adipocytes^36^. MHC II-deficient mice exhibit less adipose tissue inflammation and are more insulin-sensitive when they are fed a HFD. In WAT of PWK, both MHC I and MHC II were down-regulated, suggesting that MHC I is also associated with HFD-induced obesity. In addition to MHC I and MHC II, other factors may be involved; further studies are needed to elucidate the mechanisms underlying the superior resistance to obesity and glucose clearance in PWK mice.

Among the 438 up-regulated genes, *Ucp1* expression in WAT was highly up-regulated (378-fold) in PWK relative to B6. The UCP1 protein, which is mainly expressed in BAT, plays roles in thermogenesis, regulation of energy expenditure, and protection against oxidative stress. However, UCP1-expressing cells are not strictly limited to BAT, but are also interspersed in WAT. Like brown adipocytes, brite or beige adipocyte can also act in thermogenesis and heat production, leading to obesity resistance^37^. A previous report showed that transgenic expression of UCP1 in fat increases oxygen consumption in BAT and WAT and reduces body weight gain^38^. In WAT of PWK, several brown or beige markers, including Lhx8, CD137, Klh13, and Tmem26, were up-regulated relative to WAT of B6, suggesting that WAT of PWK might have characteristics of brite adipocytes, resulting in up-regulation of thermogenesis and increased energy expenditure. Consequently, PWK mice exhibit superior resistance to obesity and glucose clearance relative to B6 mice.

## Materials and Methods

### Ethics statement

All animal experiments were approved by the Animal Care and Experimentation Committee, Gunma University, Showa Campus, Japan, and were conducted according to the guidelines of this committee.

### Animals and diets

C57BL/6J (B6) mice were purchased from Charles River Japan. PWK mice were purchased from RIKEN BioResource Center, Tsukuba, Japan. Mice were housed in box cages and maintained on a 12-h light/12-h dark cycle. B6 and PWK male mice and F1 male progeny obtained by reciprocal crosses between them were placed in single cages and fed either a normal diet (Research Diets, New Brunswick, NJ, USA, catalog [cat.] #D06072701) or a HFD (Research Diets, #D07012601) for 15 weeks, starting at the age of 6 weeks.

### RNA preparation

Total RNA was prepared from isolated whole gonadal adipose tissues using the AllPrep DNA/RNA mini kit (Qiagen, Hilden, Germany).

### Library preparation, sequencing, and data analysis

RNA quality was assessed using an Agilent RNA 6000 Nano Kit (Agilent, Palo Alto, CA, USA, cat. #5067-1511) on an Agilent 2100 Bioanalyzer. The RNA was prepared for sequencing using the TruSeq RNA sample preparation kit v2 Set B (Illumina, San Diego, CA, USA, cat. #RS-122-2002). Each sample was prepared using 1 μg of total RNA. The resulting cDNA libraries were quantified using the KAPA Library Quantification kit (KAPA Biosynthesis, Woburn, MA, USA, cat. #KK4835), and quality and size were checked using the Agilent High Sensitivity DNA Kit (Agilent, cat. #5067-4626). The average fragment length of the cDNA libraries was 280 bp. The samples were loaded onto a cBot (Illumina) for clustering in a flow cell, and the flow cell was then sequenced on a HiSeq 1000 (Illumina). A paired-end (2 × 101) run was performed using the SBS kit (Illumina, cat. #FC-401-3001). Real-time analysis and base calling were performed using the HiSeq Control Software Version 1.5 (Illumina). A summary of the reads in each sample is shown in Table S1. Alignment to the mouse genome (mm9) was performed using TopHat (http://tophat.cbcb.umd.edu/). All aligned reads were exported in BAM format, and subsequent data analysis was performed using Avadis NGS (Strand Scientific Intelligence, San Francisco, CA, USA). RNA expression analysis was performed at the exon level, and expression data were normalized with trimmed mean of M values (TMM)^39^. The normalized counts were log-transformed and baselined to median of all samples. The expression levels of 15825 genes were analyzed to identify differentially expressed genes (moderate t-test, p < 0.05, >2-fold). Functional annotation of differentially expressed genes was performed with DAVID (http://david.abcc.ncifcrf.gov/)^9^,^10^.

A list of imprinted mouse genes can be downloaded from the Geneimprint web site (http://www.geneimprint.com/site/genes-by-species.Mus+musculus). The *p*-value of the difference in expression (HFD - Con) was calculated using Student’s *t*-test (p < 0.05). Imprinted genes with significantly different expression levels were identified using Student’s *t*-test (p < 0.05). The *p*-values of down-regulated PEGs or up-regulated MEGs were calculated using the chi-square test.

### Screening for new imprinted genes in WAT

Allelic expression was examined using SNPs that differ between B6 and PWK detected in our RNA-seq data. Both F1 mice of reciprocal cross between B6 and PWK, (B6 × PWK) F1 and (PWK × B6) F1, were used to avoid any effects of allelic bias. Using the AVADIS NGS software, SNPs that differ between B6 and PWK were extracted. After excluding SNPs on the Y chromosome, SNPs not in identified exons, and SNPs with a small number of reads (average total number of reads <10, at least one mouse with no reads), the remaining SNPs were used for further screening of imprinted genes. For strict screening of PEGs (MEGs), genes with at least one SNP for which the percentage of paternal (maternal) allele expression was over 99% in both (B6 × PWK) F1 and (PWK × B6) F1 were selected. For mild screening of PEGs, genes with at least four SNPs for which the percentage of the allelic expression satisfied the following conditions were selected: (1) PWK allele expression in (B6 × PWK) F1 was significantly higher than that in (PWK × B6) F1 (Student’s t-test, p < 0.05), (2) PWK allele expression in (B6 × PWK) F1 was over 50% and that in (PWK × B6) F1 was under 50%, and (3) PWK allele expression in (B6 × PWK) F1 was at least 20% higher than that in (PWK × B6) F1. For mild screening of MEGs, genes with at least four SNPs for which the percentage of allelic expression satisfied the following conditions were selected: (1) B6 allele expression in (B6 × PWK) F1 was significantly higher than that in (PWK × B6) F1 (Student’s t-test, p < 0.05), (2) B6 allele expression in (B6 × PWK) F1 was over 50% and that in (PWK × B6) F1 was under 50%, and (3) B6 allele expression in (B6 × PWK) F1 was at least 20% higher than that in (PWK × B6) F1.

### SNP detection by RT-PCR analysis

SNP detection by RT-PCR analysis was performed on WAT from (B6 × PWK) F1 and (PWK × B6) F1 (n = 6 for each strain/diet combination). RT-PCR was performed followed by digestion with restriction enzymes whose recognition sites contain SNPs between B6 and PWK.

The primer sequences were as follows:

Bmp3: 5′- ACT GTA TTT CTA CGT TGG AGA ATT AGT GA -3′, 5′- ATC GTT GGC GTA TAC CAA GAT ATA A -3′; and

Ces1f: 5′- GAC GGG CTC TCT CTG CAT AC -3′, 5′- ACC TCC TGC TGA CTC TCC AA -3′

The PCR products were digested with MboI (Bmp3) and BtsMutI (Ces1f) to discriminate between B6 and PWK alleles.

## Additional Information

**Accession Codes**: These data were deposited in the DDBJ Sequence Read Archive (DRA001186).

**How to cite this article**: Morita, S. *et al.* Gene expression profiling of white adipose tissue reveals paternal transmission of proneness to obesity. *Sci. Rep.* **6**, 21693; doi: 10.1038/srep21693 (2016).

## Supplementary Material

Supplementary Dataset

## Figures and Tables

**Table 1:** GO analysis of B6 paternal allele-dependent up-regulated (a) or down-regulated (b) genes.

Category	Term	p-value	Benjamini
(a) Genes up-regulated (p < 0.05, >2.0-fold) in WAT of B6 and (PWK×B6) F1 (B6 paternal allele-dependent up-regulated genes)			
GOTERM_BP_FAT	GO:0001775 ~ cell activation	4.99E-08	6.22E-05
GOTERM_BP_FAT	GO:0045321 ~ leukocyte activation	3.54E-07	2.21E-04
GOTERM_BP_FAT	GO:0006955 ~ immune response	5.43E-07	2.25E-04
GOTERM_BP_FAT	GO:0002684 ~ positive regulation of immune system process	5.43E-06	1.69E-03
GOTERM_BP_FAT	GO:0009611 ~ response to wounding	6.09E-06	1.52E-03
GOTERM_BP_FAT	GO:0002274 ~ myeloid leukocyte activation	7.30E-06	1.52E-03
GOTERM_BP_FAT	GO:0050865 ~ regulation of cell activation	1.03E-05	1.83E-03
GOTERM_BP_FAT	GO:0045576 ~ mast cell activation	4.21E-05	6.54E-03
GOTERM_BP_FAT	GO:0006952 ~ defense response	4.68E-05	6.45E-03
GOTERM_BP_FAT	GO:0002694 ~ regulation of leukocyte activation	5.30E-05	6.59E-03
(b) Genes down-regulated (p < 0.05, >2.0-fold) in WAT of B6 and (PWK×B6) F1 (B6 paternal allele- dependent down-regulated genes)			
GOTERM_BP_FAT	GO:0015672 ~ monovalent inorganic cation transport	3.43E-06	5.49E-03
GOTERM_BP_FAT	GO:0030001 ~ metal ion transport	1.06E-05	8.42E-03
GOTERM_BP_FAT	GO:0006811 ~ ion transport	4.40E-05	2.32E-02
GOTERM_BP_FAT	GO:0006814 ~ sodium ion transport	4.45E-05	1.77E-02
GOTERM_BP_FAT	GO:0006812 ~ cation transport	4.54E-05	1.44E-02
GOTERM_BP_FAT	GO:0001501 ~ skeletal system development	6.85E-05	1.81E-02
GOTERM_BP_FAT	GO:0048562 ~ embryonic organ morphogenesis	1.03E-04	2.32E-02
GOTERM_BP_FAT	GO:0048568 ~ embryonic organ development	1.13E-04	2.24E-02
GOTERM_BP_FAT	GO:0060348 ~ bone development	1.36E-04	2.38E-02
GOTERM_BP_FAT	GO:0001503 ~ ossification	2.88E-04	4.51E-02
GOTERM_CC_FAT	GO:0042995 ~ cell projection	8.74E-08	2.23E-05
GOTERM_CC_FAT	GO:0005929 ~ cilium	3.89E-07	3.31E-05
GOTERM_CC_FAT	GO:0044463 ~ cell projection part	3.18E-05	1.62E-03
GOTERM_CC_FAT	GO:0044441 ~ cilium part	3.28E-05	1.39E-03

**Table 2:** GO analysis of B6 maternal allele-dependent up-regulated genes.

Category	Term	p-value	Benjamini
GOTERM_BP_FAT	GO:0007049 ~ cell cycle	2.55E-14	3.45E-11
GOTERM_BP_FAT	GO:0000279 ~ M phase	1.42E-10	9.62E-08
GOTERM_BP_FAT	GO:0022403 ~ cell cycle phase	2.97E-10	1.34E-07
GOTERM_BP_FAT	GO:0000278 ~ mitotic cell cycle	6.32E-10	2.14E-07
GOTERM_BP_FAT	GO:0000087 ~ M phase of mitotic cell cycle	1.18E-09	3.20E-07
GOTERM_BP_FAT	GO:0022402 ~ cell cycle process	1.38E-09	3.11E-07
GOTERM_BP_FAT	GO:0051301 ~ cell division	6.02E-09	1.16E-06
GOTERM_BP_FAT	GO:0007067 ~ mitosis	7.29E-09	1.23E-06
GOTERM_BP_FAT	GO:0000280 ~ nuclear division	7.29E-09	1.23E-06
GOTERM_BP_FAT	GO:0048285 ~ organelle fission	1.19E-08	1.79E-06

Genes up-regulated (p < 0.05, >2.0-fold) in WAT of B6 and (B6×PWK) F1 (B6 maternal allele dependent up-regulated genes).

**Table 3:** Candidate imprinted genes.

PEG candidates			
Gene symbol	Chr	Location	Description
Aldh4a1	4	139178809..139205606	aldehyde dehydrogenase 4 family, member A1
Ank2	3	126624525..126701370	ankyrin 2, brain
Arhgef37	18	61653448..61696190	Rho guanine nucleotide exchange factor (GEF) 37
Atp8b1	18	64688633..64820654	ATPase, class I, type 8B, member 1
Bid	6	120843137..120866838	BH3 interacting domain death agonist
Bmp3	5	99283458..99309979	bone morphogenetic protein 3
Cd44	2	102651298..102741822	CD44 antigen
Chst11	10	82448242..82658645	carbohydrate sulfotransferase 11
Galntl1	12	81619977..81704883	UDP-N-acetyl-alpha-D-galactosamine:polypeptide N-acetylgalactosaminyltransferase 16
Lrrn4cl	19	8925275..8928399	LRRN4 C-terminal like
Mrc2	11	105153960..105212459	mannose receptor, C type 2
Nin	12	71112422..71212912	ninein
Paqr7	4	134052893..134064725	progestin and adipoQ receptor family member VII
Prr5l	2	101554442..101679137	proline rich 5 like
Slc16a12	19	34742896..34821601	solute carrier family 16 (monocarboxylic acid transporters), member 12
Slc5a3	16	92058567..92087718	solute carrier family 5 (inositol transporters), member 3
Slc7a5	8	124405046..124431586	solute carrier family 7 (cationic amino acid transporter, y+ system), member 5
Tbc1d8	1	39428340..39535592	TBC1 domain family, member 8
Tmem38b	4	53838917..53874890	transmembrane protein 38B
Trim16	11	62633755..62656450	tripartite motif-containing 16
Vps37b	5	124454650..124482269	vacuolar protein sorting 37B (yeast)
Zfp871	17	32902441..32925232	zinc finger protein 871
**MEG candidates**			
Acss1	2	150443847..150493976	acyl-CoA synthetase short-chain family member 1
Ces1f	8	95780135..95803635	carboxylesterase 1F
Cobl	11	12136679..12364963	cordon-bleu WH2 repeat
Dennd1b	1	140860286..141072620	DENN/MADD domain containing 1B
Myo1b	1	51806609..51972818	myosin IB
P2ry2	7	108145086..108160505	purinergic receptor P2Y, G-protein coupled 2
Parvg	15	84155150..84173408	parvin, gamma
Prkab2	3	97462135..97477006	protein kinase, AMP-activated, beta 2 non-catalytic subunit
Slc16a9	10	69708024..69748699	solute carrier family 16 (monocarboxylic acid transporters), member 9
Slc4a4	5	89316285..89668681	solute carrier family 4 (anion exchanger), member 4
Vash1	12	88019650..88036631	vasohibin 1

**Table 4:** GO analysis of down-regulated genes in WAT of PWK fed HFD or control diet relative to B6 fed a control diet.

Category	Term	p-value	Benjamini
GOTERM_CC_FAT	GO:0005576 ~ extracellular region	8.10E-10	1.80E-07
GOTERM_CC_FAT	GO:0005886 ~ plasma membrane	4.03E-06	4.47E-04
GOTERM_CC_FAT	GO:0044459 ~ plasma membrane part	2.10E-05	1.55E-03
GOTERM_CC_FAT	GO:0031226 ~ intrinsic to plasma membrane	3.99E-04	2.19E-02
GOTERM_CC_FAT	GO:0009897 ~ external side of plasma membrane	7.93E-04	3.46E-02
GOTERM_CC_FAT	GO:0005887 ~ integral to plasma membrane	1.07E-03	3.35E-02

## References

[b1] Frontera M., Dickins B., Plagge A. & Kelsey G. Imprinted genes, postnatal adaptations and enduring effects on energy homeostasis. Adv. Exp. Med. Biol. 626, 41–61 (2008). 18372790 10.1007/978-0-387-77576-0_4

[b2] Dong C. *et al.* Possible genomic imprinting of three human obesity-related genetic loci. Am. J. Hum. Genet. 76, 427–437 (2005). 15647995 10.1086/428438 PMC1196395

[b3] Gorlova O. Y. *et al.* Genetic linkage and imprinting effects on body mass index in children and young adults. Eur. J. Hum. Genet. 11, 425–432 (2003). 12774034 10.1038/sj.ejhg.5200979

[b4] Lindsay R. S., Kobes S., Knowler W. C., Bennett P. H. & Hanson R. L. Genome-wide linkage analysis assessing parent-of-origin effects in the inheritance of type 2 diabetes and BMI in Pima Indians. Diabetes, 50, 2850–2857 (2001). 11723070 10.2337/diabetes.50.12.2850

[b5] Rance K. A. *et al.* A paternally imprinted QTL for mature body mass on mouse chromosome 8. Mamm. Genome 16, 567–577 (2005). 16180138 10.1007/s00335-005-0012-4

[b6] Mantey C., Brockmann G. A., Kalm E. & Reinsch N. Mapping and exclusion mapping of genomic imprinting effects in mouse F2 families. J. Hered. 96, 329–338 (2005). 15761081 10.1093/jhered/esi044

[b7] Cheverud J. M. *et al.* Genomic imprinting effects on adult body composition in mice. Proc. Natl. Acad. Sci. USA 105, 4253–4258 (2008). 18337500 10.1073/pnas.0706562105 PMC2393747

[b8] Morita S. *et al.* Paternal allele influences high fat diet-induced obesity. PLoS One. 4, 9(1), e85477 (2014). 24416415 10.1371/journal.pone.0085477 PMC3885714

[b9] Huang D. W., Sherman B. T. & Lempicki R. A. Systematic and integrative analysis of large gene lists using DAVID Bioinformatics Resources. Nature Protoc. 4, 44–57 (2009). 19131956 10.1038/nprot.2008.211

[b10] Huang D. W., Sherman B. T. & Lempicki R. A. Bioinformatics enrichment tools: paths toward the comprehensive functional analysis of large gene lists. Nucleic Acids Res. 37, 1–13 (2009). 19033363 10.1093/nar/gkn923 PMC2615629

[b11] Klingenspor M. Cold-induced recruitment of brown adipose tissue thermogenesis. Exp. Physiol. 88, 141–148 (2003). 12525862 10.1113/eph8802508

[b12] Petrovic N. *et al.* Chronic peroxisome proliferator-activated receptor gamma (PPARgamma) activation of epididymally derived white adipocyte cultures reveals a population of thermogenically competent, UCP1-containing adipocytes molecularly distinct from classic brown adipocytes. J. Biol. Chem. 285, 7153–7164 (2010). 20028987 10.1074/jbc.M109.053942 PMC2844165

[b13] Wu J. *et al.* Beige adipocytes are a distinct type of thermogenic fat cell in mouse and human. Cell 150, 366–376 (2012). 22796012 10.1016/j.cell.2012.05.016 PMC3402601

[b14] Cinti S. *et al.* Adipocyte death defines macrophage localization and function in adipose tissue of obese mice and humans. J. Lipid. Res. 46, 2347–2355 (2005). 16150820 10.1194/jlr.M500294-JLR200

[b15] Shoelson S. E., Lee J. & Goldfine A. B. Inflammation and insulin resistance. J. Clin. Invest. 116, 1793–1801 (2006). 16823477 10.1172/JCI29069 PMC1483173

[b16] Heilbronn L. K. & Campbell L. V. Adipose tissue macrophages, low grade inflammation and insulin resistance in human obesity. Curr. Pharm. Des. 14, 1225–1230 (2008). 18473870 10.2174/138161208784246153

[b17] Oliver E., McGillicuddy F., Phillips C., Toomey S. & Roche H. M. The role of inflammation and macrophage accumulation in the development of obesity-induced type 2 diabetes mellitus and the possible therapeutic effects of long-chain n-3 PUFA. Proc. Nutr. Soc. 69, 232–243 (2010). 20158940 10.1017/S0029665110000042

[b18] Cildir G., Akıncılar S. C. & Tergaonkar V. Chronic adipose tissue inflammation: all immune cells on the stage. Trends Mol. Med. 19, 487–500 (2013). 23746697 10.1016/j.molmed.2013.05.001

[b19] Pazour G. J. & Witman G. B. The vertebrate primary cilium is a sensory organelle. Curr. Opin. Cell Biol. 15, 105–110 (2003). 12517711 10.1016/s0955-0674(02)00012-1

[b20] Singla V. & Reiter J. F. The primary cilium as the cell’s antenna: signaling at a sensory organelle. Science 313, 629–633 (2006). 16888132 10.1126/science.1124534

[b21] Suh J. M. *et al.* Hedgehog signaling plays a conserved role in inhibiting fat formation. Cell Metab. 3, 25–34 (2006). 16399502 10.1016/j.cmet.2005.11.012

[b22] Cousin W., Fontaine C., Dani C. & Peraldi P. Hedgehog and adipogenesis: fat and fiction. Biochimie, 89, 1447–1453 (2007). 17933451 10.1016/j.biochi.2007.08.012

[b23] Kim J. C. *et al.* The Bardet-Biedl protein BBS4 targets cargo to the pericentriolar region and is required for microtubule anchoring and cell cycle progression. Nat. Genet. 36, 462–470 (2004). 15107855 10.1038/ng1352

[b24] Nachury M. V. *et al.* A core complex of BBS proteins cooperates with the GTPase Rab8 to promote ciliary membrane biogenesis. Cell, 129, 1201–1213 (2007). 17574030 10.1016/j.cell.2007.03.053

[b25] Stoetzel C. *et al.* Identification of a novel BBS gene (BBS12) highlights the major role of a vertebrate-specific branch of chaperonin-related proteins in Bardet-Biedl syndrome. Am. J. Hum. Genet. 80, 1–11 (2007). 17160889 10.1086/510256 PMC1785304

[b26] Hearn T. *et al.* Subcellular localization of ALMS1 supports involvement of centrosome and basal body dysfunction in the pathogenesis of obesity, insulin resistance, and type 2 diabetes. Diabetes 54, 1581–1587 (2005). 15855349 10.2337/diabetes.54.5.1581

[b27] Aguilar V. & Fajas L. Cycling through metabolism. EMBO Mol. Med. 2, 338–348 (2010). 20721988 10.1002/emmm.201000089 PMC3118222

[b28] Haig D. Genetic conflicts in human pregnancy. Q. Rev. Biol. 68, 495–532 (1993). 8115596 10.1086/418300

[b29] Haig D. Parental antagonism, relatedness asymmetries, and genomic imprinting. Proc. Biol. Sci. 264, 1657–1662 (1997). 9404029 10.1098/rspb.1997.0230 PMC1688715

[b30] Wilkins J. F. & Haig D. What good is genomic imprinting: the function of parent-specific gene expression. Nat. Rev. Genet. 4, 359–368 (2003). 12728278 10.1038/nrg1062

[b31] Haig D. Genomic imprinting and kinship: how good is the evidence? Annu. Rev. Genet. 38, 553–585 (2004). 15568986 10.1146/annurev.genet.37.110801.142741

[b32] Haig D. Coadaptation and conflict, misconception and muddle, in the evolution of genomic imprinting. Heredity (Edinb) 113, 96–103 (2014). 24129605 10.1038/hdy.2013.97 PMC4105449

[b33] Haig D. & Wharton R. Prader-Willi syndrome and the evolution of human childhood. Am. J. Hum. Biol. 15, 320–329 (2003). 12704708 10.1002/ajhb.10150

[b34] Mott R. *et al.* The architecture of parent-of-origin effects in mice. Cell, 156, 332–342 (2014). 24439386 10.1016/j.cell.2013.11.043 PMC3898482

[b35] Kobayashi H. *et al.* Contribution of intragenic DNA methylation in mouse gametic DNA methylomes to establish oocyte-specific heritable marks. Plos Genet. 8, e1002440 (2012). 22242016 10.1371/journal.pgen.1002440 PMC3252278

[b36] Deng T. *et al.* Class II major histocompatibility complex plays an essential role in obesity-induced adipose inflammation. Cell Metab. 17, 411–422 (2013). 23473035 10.1016/j.cmet.2013.02.009 PMC3619392

[b37] Shabalina I. G. *et al.* UCP1 in brite/beige adipose tissue mitochondria is functionally thermogenic. Cell Rep. 5, 1196–1203 (2013). 24290753 10.1016/j.celrep.2013.10.044

[b38] Kopecky J., Clarke G., Enerbäck S., Spiegelman B. & Kozak L. P. Expression of the mitochondrial uncoupling protein gene from the aP2 gene promoter prevents genetic obesity. J. Clin. Invest. 96, 2914–23 (1995). 8675663 10.1172/JCI118363 PMC186003

[b39] Robinson M. D. & Oshlack A. A scaling normalization method for differential expression analysis of RNA-seq data. Genome Biol. 11, R25 (2011). 20196867 10.1186/gb-2010-11-3-r25 PMC2864565

